# The role of patent waivers and compulsory licensing in facilitating access to COVID-19 vaccines: Findings from a survey among healthcare practitioners in Nigeria

**DOI:** 10.1371/journal.pgph.0000683

**Published:** 2022-07-07

**Authors:** Obi Peter Adigwe, Davidson Oturu

**Affiliations:** 1 National Institute for Pharmaceutical Research and Development, Abuja, Federal Capital Territory, Nigeria; 2 Aelex, Abuja, Federal Capital Territory, Nigeria; University of Ottawa, CANADA

## Abstract

The roll out of COVID-19 vaccines has again revealed the gap between high income countries and developing nations in terms of access to healthcare commodities and services. With the slow vaccination roll out in many low income countries and the emergence of more contagious variants of COVID-19, many persons are at risk of contracting the disease in settings with low immunisation coverage. This study aimed at exploring the views of healthcare practitioners on the role of patent waivers and compulsory licensing in facilitating access to vaccines. A cross-sectional study was undertaken among practitioners in the health sector, which comprised private, public, and development agencies. A well structured and validated questionnaire was administered to the study participants using both physical and online methods of administration in Nigerian setting. A total of 526 respondents participated in the study, majority of them were males (54.4%). A third of the study participants (31.1%) had postgraduate degrees. A strong majority of the respondents (81.2%) agreed that the role of patent is to promote innovation, whilst 70.6% of them indicated that intellectual property waivers can improve access to COVID-19 vaccines. Slightly above half of the respondents (56.0%) indicated that patent waivers can reduce innovation in the pharmaceutical sector, they however indicated that such challenge can be mitigated by granting incentives to innovators whose intellectual property rights had been waived. This study has revealed that there is a need for intellectual property rights waiver and compulsory licensing of all novel COVID-19 commodities including vaccines, as this is an important strategy that can improve access to relevant products in developing countries.

## Introduction

Despite being the “saviours of public health”, pharmaceutical companies are still commercial entities that depend on intellectual property to recoup their investment on research and development carried out. Indeed, intellectual property has always been the backbone of the pharmaceutical industry [1], and this has been obvious in recent years. Firstly, new medicines that are discovered are protected as patents. When such discovery is ready for circulation to the public, the manufacturer then gives it a name, which is then protected as a trademark. These intellectual property rights allow the manufacturers to have exclusivity over the use of their intellectual property for a number of years [2]. Thus, where manufacturers fail to leverage on these intellectual property rights, they may be unable to make profit out of their inventions [3]. However, sometimes the patents that grant exclusivity may have to be mitigated, especially when the patents under consideration holds the key to nations escaping the ongoing pandemic.

By the late twentieth century, technology and innovation had begun to advance [4], and this meant that devices which could reproduce creations at a relatively low cost were being produced. Industries that were heavily reliant on intellectual property protection for generation of their revenue saw their works being pirated by companies in other jurisdictions and being sold at lower prices [5], consequently pushing the original products out of the market in the infringer’s country. These and other factors led to the introduction of Trade-Related Aspects of Intellectual Property Rights (TRIPS) agreement [6–8], which is a multilateral agreement aimed at reducing distortions and impediments to international trade, whilst taking into account the need to promote effective protection of intellectual property rights and ensuring that they do not themselves become a barrier to international trade. At the signing of the TRIPS agreement, any nation that wanted to take part in the World Trade Organisation (WTO) was obliged to amend its intellectual property legislation to meet the guidelines set out in the agreement, thus creating a uniform international standard of protection for intellectual property rights. This set out a standard system for intellectual property which was able to protect inventions across borders.

In Nigeria and many other developing countries, Medicines’ Security has been a challenge over the years [9], and the emergence of COVID-19 has further affected access to healthcare commodities [10]. The world has undergone drastic changes as a result of the COVID-19 pandemic which took nations by storm and disrupted the important facets of economy and everyday lives [11, 12]. With infections spreading and death toll rising by the day [13], there is need to ensure speedy access to COVID-19 vaccines. According to the National Primary Health Care Development Agency, as at April 4, 2022, the proportion of persons who have received first dose of COVID-19 vaccine in Nigeria is 17.4%, whilst only 11.6% of total eligible population targeted for vaccination were fully vaccinated [14]. Currently, all COVID-19 vaccines available in the country are from international sources. This study therefore aimed at assessing the role of patent waivers in facilitating access to COVID-19 vaccines.

## Methods

### Ethics statement

Prior to the commencement of data collection, ethical clearance was obtained from Health Research Ethics Committee of National Institute for Pharmaceutical Research and Development with approval number NHREC/039/21A. Written informed consent was obtained from physical respondents before administering the questionnaires to them. Whilst for those who responded online, informed consent form was sent along with the electronic copy of the questionnaire, and only persons who gave their consent to participate could proceed to the next step of filling the questionnaire. Confidentiality was strictly maintained during the data collection process.

### Study design

A cross-sectional study was undertaken in Abuja between June and July 2021. The study was carried out among healthcare practitioners that participated in a conference targeted at improving access to vaccines in Nigeria and other developing countries. The practitioners include those in private sector, public sector, and development agencies.

### Study tool

The questionnaire for the study was designed in English language following extensive literature review [15–17], and was made up of demography and section relating to intellectual property rights and vaccines access (S1 Text). A Likert type scale of 1 to 5 was used to assess the views and perspectives of the participants, and the coding was as follows: 1 = strongly disagree, 2 = disagree, 3 = neutral, 4 = agree, and 5 = strongly agree. The research instrument was then validated by a team of experts and pretested on 25 participants. The feedback received did not necessitate any major change.

### Data collection

A convenience sampling approach was adopted for data collection [18]. Paper questionnaires were administered to all participants physically present, whilst an online questionnaire was generated using Google Forms and sent to emails of those that took part in the conference remotely. A follow up reminder was sent to online participants in order to increase the response rate.

Data were entered into Statistical Package for Social Sciences software version 25 after data collection. Descriptive and inferential statistical analyses were undertaken. A *p* value of 0.05 or less was considered as threshold for statistical significance.

## Results

### Demography

Of the 605 participants that received the questionnaire, a total of 526 valid responses were received, giving a response rate of 86.94%. Majority of the participants were males (54.4%), the dominant age group was 31 to 40 years (30.8%), a tenth of the participants (10.6%) were educated up to doctorate level. Further details on socio demographic characteristics are presented in Table 1 below.

**Table 1:** Socio demographic characteristics of participants.

Variable	Frequency (%)
Gender	
Male	286 (54.4)
Female	240 (45.6)
Age (years)	
≤ 30	145 (27.6)
31–40	162 (30.8)
41–50	91 (17.3)
51–60	108 (20.5)
Above 60	20 (3.8)
Highest Educational Level	
National Diploma/NCE	72 (13.9)
First Degree/HND	285 (55.0)
Masters’ Degree	106 (20.5)
PhD	55 (10.6)
Occupation	
Government Sector	223 (43.6)
Private Sector	204 (39.8)
Development Agency	45 (8.8)
Others	22 (4.3)

Note: HND = Higher National Diploma, NCE = National Certificate of Education

### Role of patent

**Fig 1:**
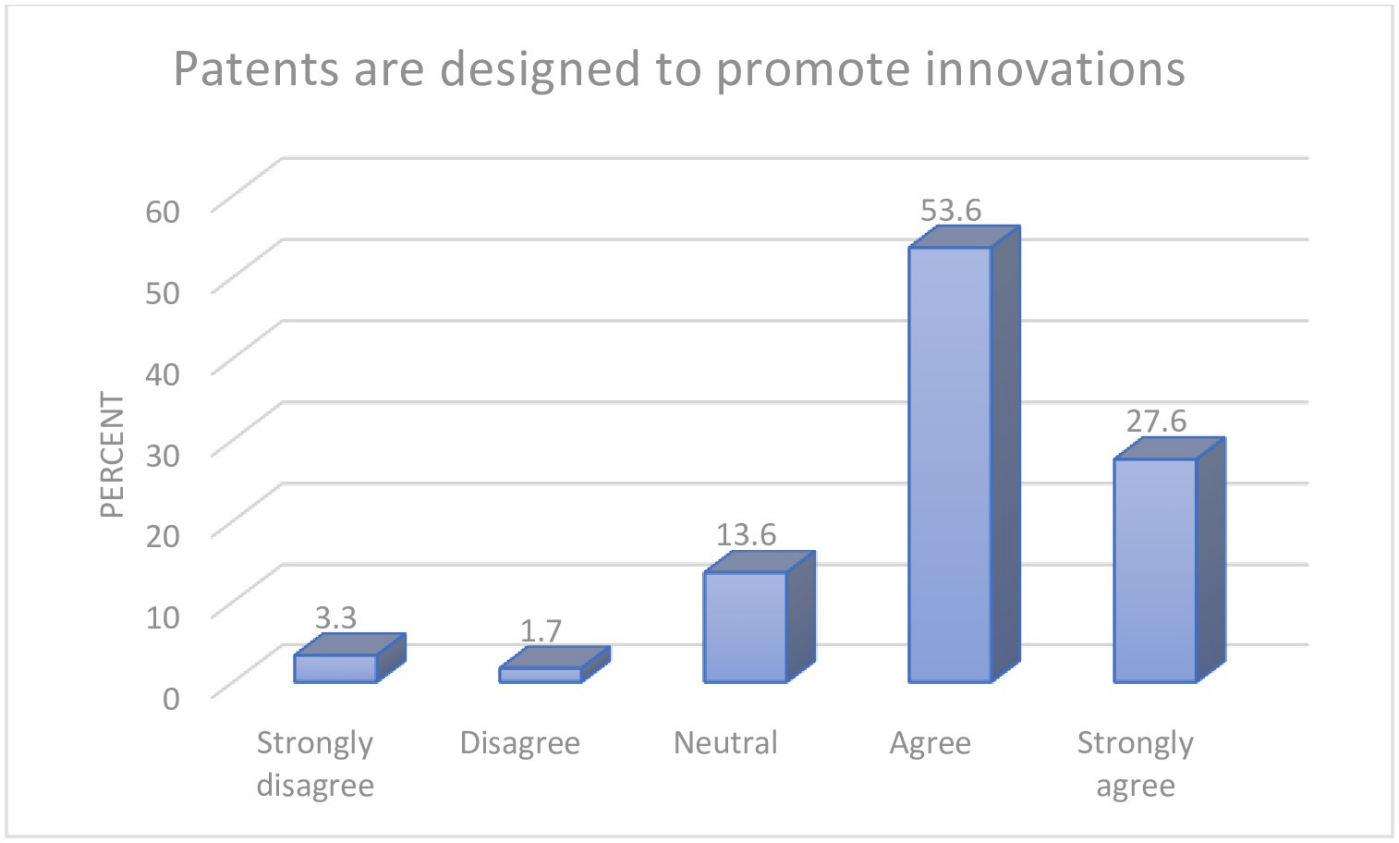
Knowledge on role of patents.

A strong majority of the participants (81.2%) agreed that the role of patents is to promote innovations. Only 5% of the respondents disagreed with this perspective, whilst 13.6% of the remaining participants were neutral in their response. Further details are presented in Fig 1.

The finding in Fig 1 indicates that the participants were familiar with the objectives of patent.

### Role of intellectual property rights waiver on access to vaccines

**Fig 2:**
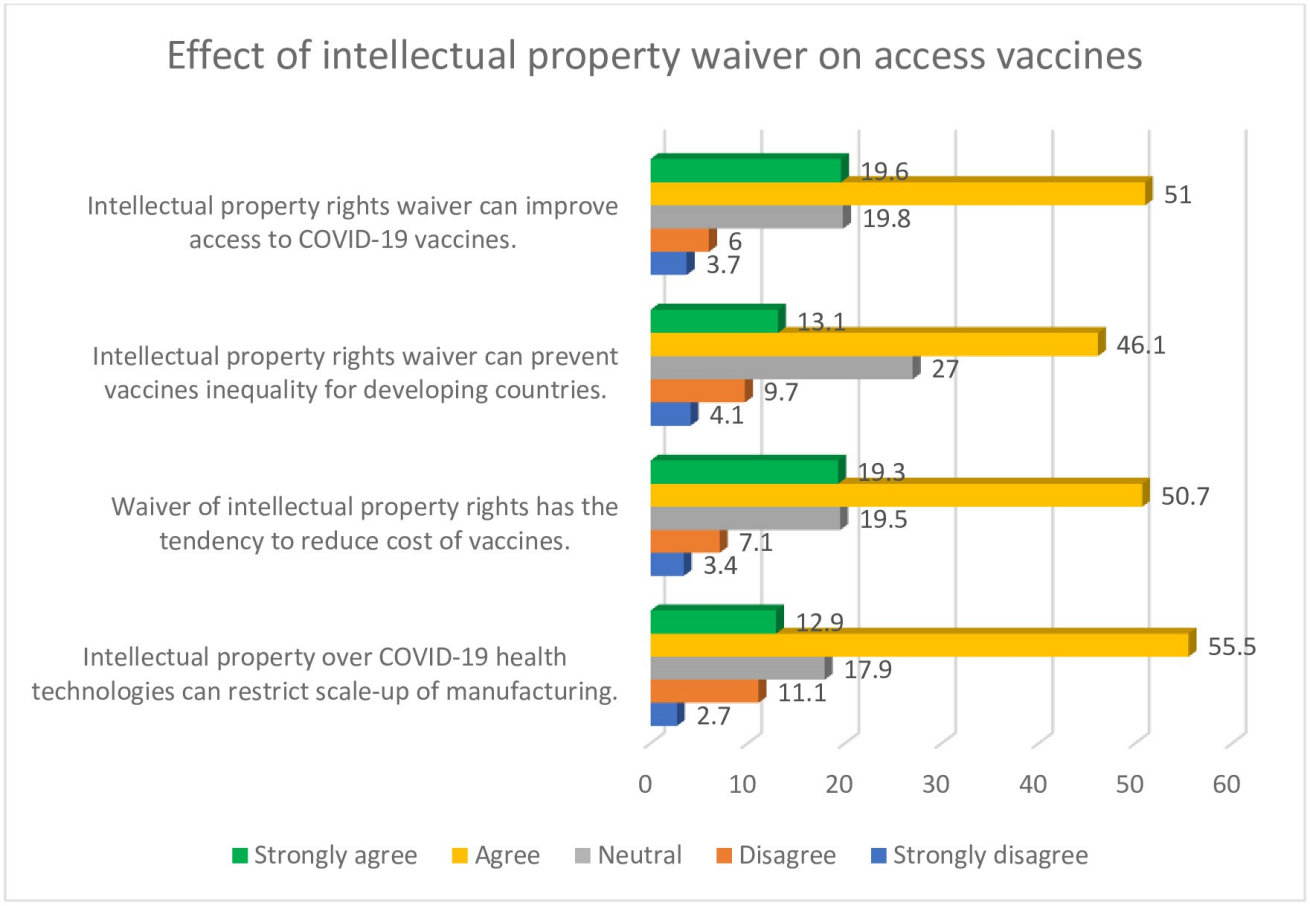
Role of intellectual property on access to vaccines.

Close to three quarters (70.6%) of the participants in this study indicated that intellectual property right waiver can improve access to COVID-19 vaccines, whilst a similar proportion (70.0%) indicated that patents waiver has the tendency to reduce cost of vaccines. Majority of the respondents (59.2%) agreed that intellectual property rights waiver can prevent vaccines inequality for developing countries. Other relevant details on role of intellectual property rights waiver on access to vaccines are provided in Fig 2.

Also, from Fig 2, slightly above two thirds of the participants (68.4%) in this study were also of the opinion that intellectual property over COVID-19 health technologies can restrict scale-up of manufacturing.

### Effect of intellectual property rights waiver on the pharmaceutical sector

**Fig 3:**
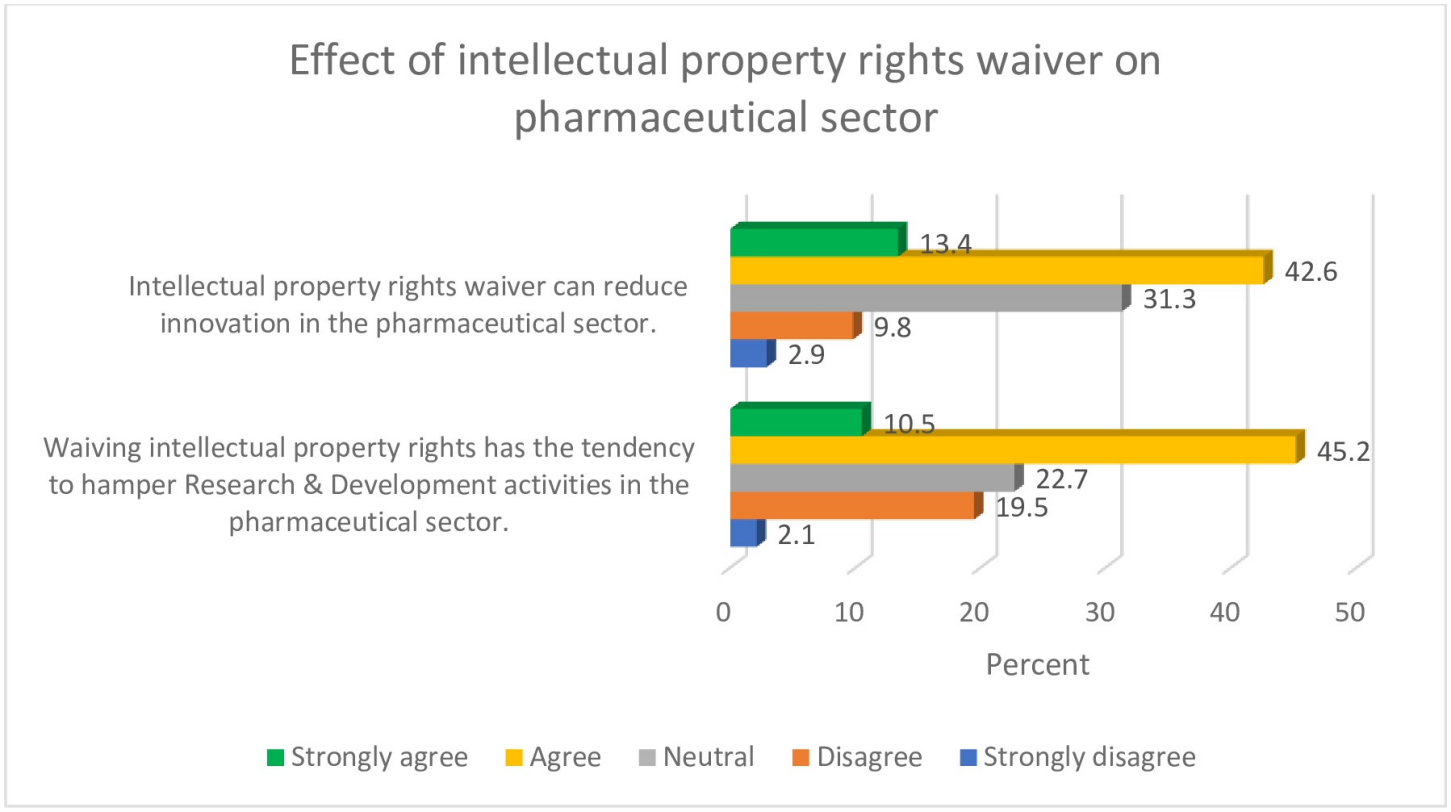
Effect of intellectual property rights waiver on pharmaceutical sector.

Findings in Fig 3 shows that more than half of the participants (56.0%) in this study were of the opinion that intellectual property rights waiver can reduce innovation in the pharmaceutical sector, whilst a third of them (31.3%) were however indifferent in this regard. Also, more than half of the respondents indicated that waiving intellectual property rights can reduce research and development activities in the sector.

Furthermore, from Fig 3, there were concerns that patents waiver can reduce innovation as well as affect research and development activities in the pharmaceutical sector.

### Incentives for intellectual property rights

**Fig 4:**
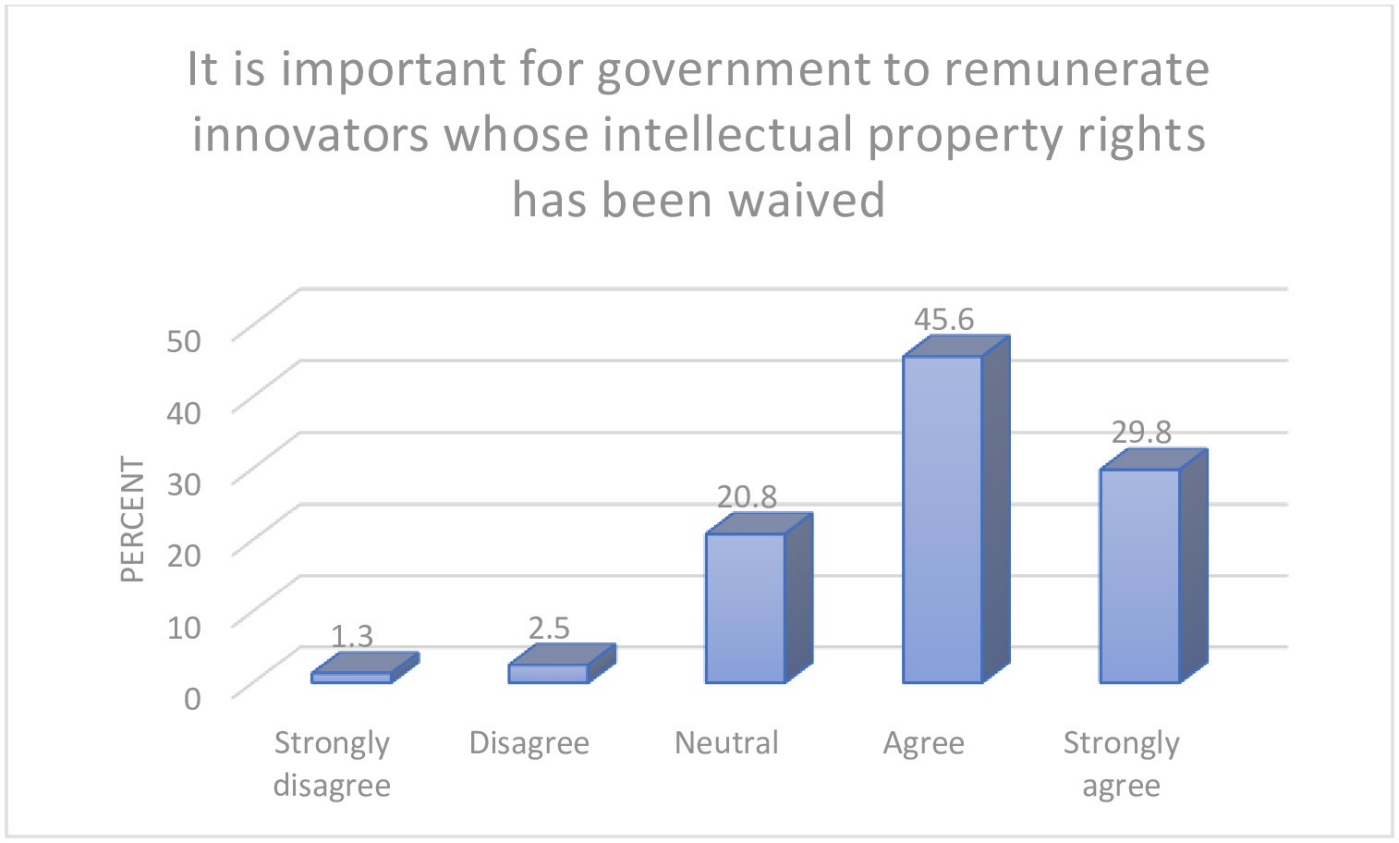
Incentives for intellectual property rights.

Three quarters of participants (75.4%) in this study indicated that it is important for government to remunerate innovators whose intellectual property rights has been waived, whilst 20.8% of the participants were undecided in this perspective. Further details are presented in Fig 4.

In addition to the descriptive statistical analysis undertaken, inferential statistical analysis was also carried out using chi square test to determine association between variables and demographic data. Findings revealed that more of the participants with master’s degrees (86.7%) and those with doctorate degrees (83.6) agreed that patents are designed to promote innovations, and more of the respondents with diplomas and first degrees were indifferent as regarding their responses in this area. These findings were statistically significant (*p*<0.001). Also, majority of the participants with doctorate degrees (54.5%) disagreed that waiver of intellectual property rights has the tendency to hamper research and development activities. This finding was also statistically significant (*p*<0.001). Further details are presented in Table 2 below.

**Table 2:** Cross tabulation of level of education with intellectual property.

Statement	Educational Level	Strongly Disagree n (%)	Disagree n (%)	Neutral n (%)	Agree n (%)	Strongly Agree n (%)	X^2^	*P-value*
Patents are designed to promote innovations	ND/NCE	2 (2.8)	1 (1.4)	14 (19.7)	50 (70.4)	4 (5.6)	63.175	<0.001
	First degree/HND	8 (2.8)	2 (0.7)	46 (16.3)	154 (54.6)	72 (25.5)		
	Master’s	1 (0.9)	3 (2.8)	10 (9.4)	54 (50.9)	38 (35.8)		
	PhD	6 (10.9)	3 (5.5)	-	19 (34.5)	27 (49.1)		
Waiving intellectual property rights has the tendency to hamper Research & Development activities in the pharmaceutical sector.	ND/NCE	1 (1.4)	7 (9.7)	29 (40.3)	31 (43.1)	4 (5.6)	79.796	<0.001
	First degree/HND	7 (2.5)	38 (13.4)	56 (19.8)	153 (54.1)	29 (10.2)		
	Master’s	3 (2.8)	26 (24.5)	23 (21.7)	41 (38.7)	13 (12.3)		
	PhD	-	30 (54.5)	7 (12.7)	9 (16.4)	9 (16.4)		

## Discussion

Although majority of the participants in this study agreed that patents are targeted at promoting inventions, their responses appear to be affected by their level of education. The respondents were however in consensus that waiving intellectual property rights can increase access to COVID-19 vaccines. This is an important finding to help address vaccine inequality which has occurred as a result of vaccine nationalism and scramble by developed countries [19]. TRIPS agreement was made in order to protect intellectual property rights, however, it was observed that the agreement had some shortcomings especially in the promotion of access to medicines. It would appear that the lacuna TRIPS agreement left was initially filled during the Doha Declaration [20, 21]. This declaration was adopted by the WTO Ministerial Conference two decades ago in Doha and it reaffirmed flexibility of TRIPS member states in circumventing patent rights for better access to essential medicines. The Doha Declaration sheds light on two mechanisms that can be used in emergencies to facilitate access to medicines, which include patent waiver and compulsory licensing of pharmaceutical products.

A considerable proportion of the sample indicated that waiver of intellectual property rights in pandemic situation can reduce cost. As the law of demand and supply states, the higher the price of goods, the lower the demand [22]. Thus, by enforcing monopolistic rights, pharmaceutical firms would exclude others from producing generic replicas of their products and consequently making the cost of pharmaceuticals unaffordable for most developing and low-income nations [23]. There must be a deviation from the orthodoxy towards the prioritisation of public health in order to flatten the curve of COVID-19 globally. In addition, local production of these biological products can help address vaccine hesitancy challenges in developing nations [24], as this can increase uptake by building more confidence.

Findings from this study suggests intellectual property over COVID-19 can restrict scale-up of manufacturing. This position is similar to previous reports [25, 26], and therefore brings the need for invoking compulsory licensing for all COVID-19 commodities especially in this critical time. Compulsory licensing means the non-voluntary acquisition of a patent from the owner combined with the payment of remuneration based on its economic value [27]. The use of compulsory licensing to address health crisis is not novel. It was used during the human immunodeficiency virus/acquired immunodeficiency syndrome (HIV/AIDS) epidemic in the early 2000s where compulsory licensing was secured in order to import generic anti-retroviral medicines by low-income nations [28]. For governments to invoke compulsory licensing provision of the TRIPS agreement to combat COVID-19, it is important that they must first domesticate these provisions or have allowances for them in their local laws. In a pandemic situation like this, prioritising the development of strategy that can save lives is not out of place. Therefore, it is important to promote policies such as suspension of patents for COVID-19 related health commodities so as to facilitate access to such products.

Following World Health Organisation declaration of the coronavirus as a global public health emergency, some countries immediately issued compulsory licences and started amending existing laws to accommodate the issuance of compulsory licences in response to the ongoing pandemic. For instance, in early 2020, the legislative arm in Canada, Chile and Ecuador began to lay the groundwork for the issuance of compulsory licences [29]. Canada in response to the pandemic amended their Patent Act to allow for a faster process in issuing compulsory licences by granting the licences first and negotiating remuneration later [30]. Chile similarly passed a resolution granting the use of compulsory licensing for the prevention and treatment of COVID-19. The resolution declares that the ongoing pandemic suffices as a national emergency to invoke compulsory licensing of COVID-19 related inventions. Additionally, in March 2020, Israel issued compulsory patent licencing for the importation of lopinavir/ritonavir, which is one of the approved testing drugs by WHO, for the management of coronavirus patients [31]. Despite all these preparations, no country has invoked compulsory licensing in relation to vaccines yet.

In this study, there were also concerns about the negative effects of waiving intellectual property rights, as slightly above half of the participants indicated that such practice could reduce innovations in the pharmaceutical sector, a third of the respondents were however indifferent in this regard. Similarly, more than half of the study participants indicated that waiving intellectual property rights has the tendency to hamper research and development activities in the pharmaceutical sector. It is important that, access to essential health commodities be prioritised so as to prevent avoidable loss of lives. Addressing concerns raised in this study is critical to improving access to essential health commodities in future pandemics. Respondents were however of the opinion that innovators whose intellectual property rights had been waived need to be remunerated by governments, and this implies that individual nation that wishes to produce items in this category locally will need to provide some forms of remuneration to the inventor before pharmaceutical companies in such countries can manufacture such products. This is an important strategy to ensure continuous innovation and research activities in the pharmaceutical sector. The critical factor for intellectual property rights waiver is to save lives. It is therefore important for governments to consider developing strategy for incentives so as to facilitate patents waiver in the pharmaceutical industry. This will serve as a way to encourage research and development activities in the health sector.

A patent waiver as the name implies means the removal of all rights accrued to an invention which normally would have granted the inventor monopoly over it [32]. Patent waivers for vaccines are also not novel as they were used during the polio epidemic. However, in this instance, the manufacturer of the vaccine voluntarily waived off the patent rights. This waiver enabled Israel to manufacture polio vaccines locally and aided them in the eradication of polio in their country. In the later parts of 2020, South Africa and India proposed at the WTO, the temporary suspension of granting patent for COVID-19 technologies. This was however rejected at the first instance by majority of developed countries. Further rejections would therefore have impact on developing countries, this is due to the fact that majority of COVID-19 vaccine intellectual property rights rests in high income countries.

Finally, findings in this study suggests that majority of healthcare practitioners supported waiver of intellectual property rights for COVID-19 vaccines and other related commodities. There is need for policymakers and WTO to develop contextual strategy to achieve this standpoint, considering the fact that these participants are important stakeholders in the healthcare sector.

## Conclusion

This study has provided new insights into the perspective of healthcare practitioners in Nigeria on role of patent waivers and compulsory licensing in facilitating access to novel commodities for COVID-19 including vaccines. Whilst there were concerns about reduction in innovation and research activities which may arise as a result of patent waivers, findings from this study also support the need for remunerating innovators whose intellectual property rights had been waived. Furthermore, it emerged that contextual policies which include certain incentives would serve as a means of reward and encouragement, and consequently stimulate innovations.

Whilst this study identified intellectual property rights waiver as a key instrument to improving supply and reducing cost of essential pharmaceutical products during pandemics, it is important for governments and policymakers to initiate robust policies underpinned by the findings from this study so as to increase responsiveness.

Given the public health importance of vaccination as well as access to essential health commodities, further studies can be undertaken so as to enable more robust and comprehensive exploration in order to deepen some of these emergent findings.

## Supporting information

S1 Text Questionnaire. (DOCX)
