# Supplementary material for: The role of patent waivers and compulsory licensing in facilitating access to COVID-19 vaccines: Findings from a survey among healthcare practitioners in Nigeria
Source: PLOS Glob Public Health. 2022 Jul 7;2(7):e0000683. doi: 10.1371/journal.pgph.0000683 (PMC10021699; doi:10.1371/journal.pgph.0000683)
Supplement: S1 Text — (DOCX) [file pgph.0000683.s001.docx]

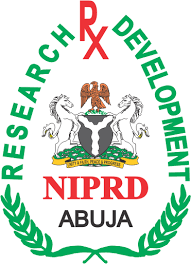
**NATIONAL INSTITUTE FOR PHARMACEUTICAL RESEARCH AND DEVELOPMENT (NIPRD) IDU, ABUJA.**

**The Role of Patent Waivers and Compulsory Licensing in Facilitating Access to COVID-19 Vaccines**

**Introduction**

Vaccine development and production is an aspect that requires critical attention so as to achieve competency in preventing vaccine preventable diseases. This questionnaire is aim at assessing your views on intellectual property rights as it relates to COVID-19 vaccines and other important COVID-19 commodities. The information you provide will be anonymised and treated confidentially.

**Section A: Demography**

1. **Gender**

| Male |  | Female |  |
| --- | --- | --- | --- |

1. **Age**

| ≤ 30 |  | 31-40 |  | 41-50 |  | 51-60 |  | Above 60 |  |
| --- | --- | --- | --- | --- | --- | --- | --- | --- | --- |

1. **Highest Educational Level**

| National Diploma/NCE |  | First Degree/HND |  | Master’s Degree |  | Doctorate Degree |  |
| --- | --- | --- | --- | --- | --- | --- | --- |

1. **Occupation**

| Government Sector |  | Private Sector |  | Development Agency |  | Retired |  | Others, please specify ………………………………. |
| --- | --- | --- | --- | --- | --- | --- | --- | --- |

**Section B: Intellectual Property Rights and Vaccine Production in Nigeria**

(To what extent do you agree or disagree with each of the following)

| **SN** | **Statement** | **Strongly Disagree** | **Disagree** | **Neutral** | **Agree** | **Strongly Agree** |
| --- | --- | --- | --- | --- | --- | --- |
| 1 | Patents are designed to promote innovations. |  |  |  |  |  |
| 2 | Intellectual property over COVID-19 health technologies can restrict scale-up of manufacturing. |  |  |  |  |  |
| 3 | Waiver of intellectual property rights has the tendency to reduce cost of vaccines. |  |  |  |  |  |
| 4 | Waiving intellectual property rights has the tendency to hamper Research & Development activities in the pharmaceutical sector. |  |  |  |  |  |
| 5 | Intellectual property rights waiver can reduce innovation in the pharmaceutical sector. |  |  |  |  |  |
| 6 | Intellectual property rights waiver can prevent vaccines inequality for developing countries. |  |  |  |  |  |
| 7 | Intellectual property rights waiver can improve access to COVID-19 vaccines. |  |  |  |  |  |
| 8 | It is important for government to remunerate innovators whose intellectual property right has been waived. |  |  |  |  |  |

1. What measures do you feel should be adopted to achieve COVID-19 vaccine production in Nigeria ............................................................................................................................................................................................
2. What other views do you have about vaccine production in Nigeria

.............................................................................................................................................................................................

**Thank you for taking time to complete this questionnaire**
